# Grapiprant: A snapshot of the current knowledge

**DOI:** 10.1111/jvp.12983

**Published:** 2021-05-31

**Authors:** Irene Sartini, Mario Giorgi

**Affiliations:** ^1^ Department of Veterinary Medicine University of Sassari Sassari Italy; ^2^ Department of Veterinary Sciences University of Pisa Pisa Italy; ^3^ PhD School Department of Veterinary Medicine University of Sassari Sassari Italy

**Keywords:** analytical method, grapiprant, pharmacodynamics, pharmacokinetics, review, safety

## Abstract

Grapiprant is the pioneer member of the novel piprant class, a potent and specific antagonist of the prostaglandin E2 receptor 4. It has been approved in veterinary medicine for the control of pain and inflammation associated with osteoarthritis in dogs at the dose regimen of 2 mg/kg once a day by the FDA and EMA (for pain only) in 2016 and 2018, respectively. The aim of this narrative review was to report the analytical methods, pharmacokinetics, pharmacodynamics and safety of grapiprant in several animal species using the best available published scientific evidence. In conclusion, most of the analytical methods proposed for grapiprant detection are simple, reliable, sensitive and validated. The pharmacokinetics show discrepancies between animal species. The therapeutic efficacy seems more suited to chronic rather than acute pain.

## 1 INTRODUCTION

In the present day, animal care management has drastically improved compared to the situation at the end of last century. Companion animals are now living longer and so are more commonly manifesting age‐related and/ or pain‐associated diseases of medical importance such as cancer, arthritis and metabolic disorders (Giorgi, 2012).

The most commonly used drugs in the treatment of acute and chronic canine pain belong to the class of non‐steroidal anti‐inflammatory drugs (NSAIDs). Nonselective or preferential NSAIDs inhibit both COX isoforms (COX‐1/COX‐2) to different extents. The second generation of NSAIDs (coxibs) is highly selective for the COX‐2 enzyme, reducing but not eliminating the risk of some adverse effects and maintaining anti‐inflammatory and analgesic actions that would alleviate inflammation and pain states. Different members of the coxib class are available for the treatment of canine OA including cimicoxib, deracoxib, firocoxib, mavacoxib and robenacoxib (Giorgi et al., 2012; Kim & Giorgi, 2013).

Osteoarthritis (OA) is a progressive condition that leads to joint inflammation, cartilage damage, pain and disability (Rychel, 2010). A number of approved NSAIDs are available for the treatment of pain associated with this chronic joint condition (Johnston et al., 2008; Kim & Giorgi, 2013; McLaughlin, 2000). The administration is usually life long and some of the above‐mentioned drugs may produce side effects. For this reason, there is the need for new drugs effective for OA pain relief with a better safety and effectiveness profiles. Grapiprant (Galliprant^®^) is an analgesic and anti‐inflammatory drug. This compound is the pioneer member of the novel piprant class. It is a potent and specific antagonist of the prostaglandin E2 (PGE2) receptor 4 (EP4). It was approved in veterinary medicine by the FDA in 2016 for the control of pain and inflammation and by the EMA in 2018 for the control of pain associated with OA in dogs (Rausch‐Derra et al., 2016). It is sold as 20, 60 and 100 mg tablets. The recommended clinical dose is 2 mg/kg once a day in fasting conditions.

## 2 PHYSICO‐CHEMICAL FEATURES

**FIGURE 1:**
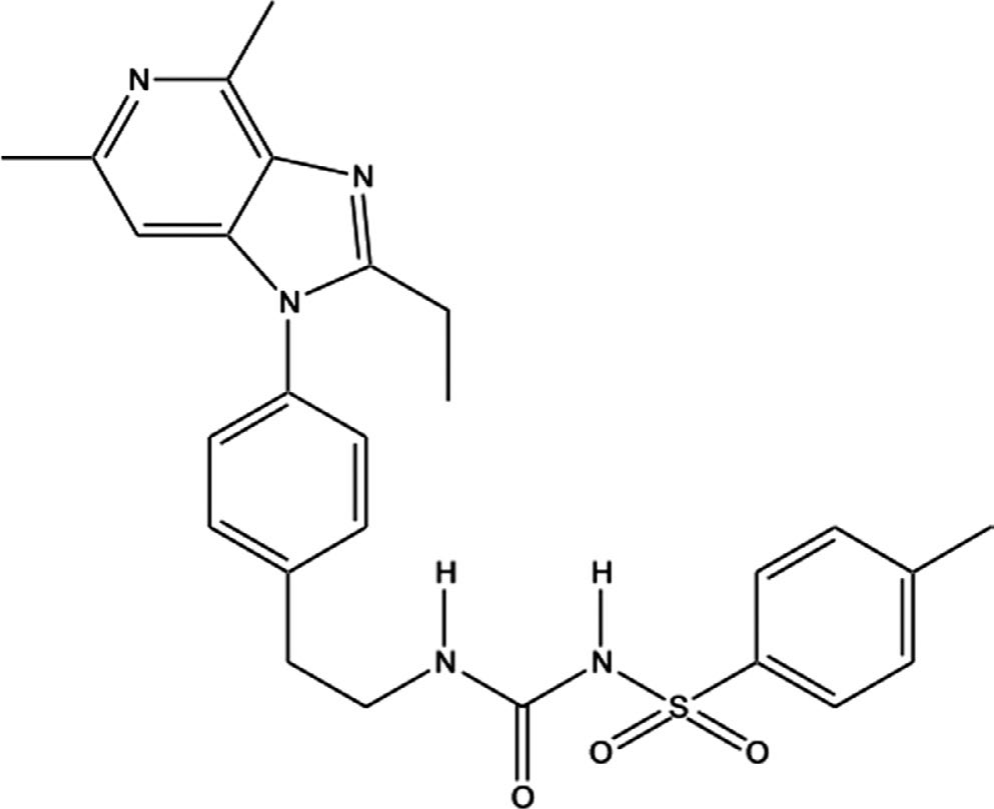
Chemical structure of grapriprant (1‐[2‐[4‐(2‐ethyl‐4,6‐dimethylimidazo[4,5‐c]pyridin‐1‐yl) phenyl]ethyl]‐3‐(4‐methylphenyl) sulfonylurea)

Grapiprant (C26H29N5O3S, 3‐[2‐(4‐{2‐ethyl‐4,6‐dimethyl‐1H‐imidazo[4,5‐c]pyridin‐1‐yl}phenyl)ethyl]‐1‐(4‐methylbenzenesulfonyl)urea) is a small molecule with a molecular weight of 491.61 g/mol. It is characterized by a logP of 4.56 and a melting point >136°C. It appears as a white to off‐white crystalline powder soluble in DMSO, but with a very poor water solubility (0.041 mg/L). Its molecular structure is depicted in Figure 1.

## 3 ANALYTICAL METHODS

A number of analytical methods for the quantification of grapiprant in different biological matrices have been published (Baralla et al., 2018; Cox et al., 2020; De Vito et al., 2016, 2017; Heit et al., 2018; Knych et al., 2018; Łebkowska‐Wieruszewska et al., 2017; Nagahisa & Okumura, 2017). The devices used for the quantification of grapiprant are HPLC and LC‐MS/MS. The sample clean up mainly uses liquid‐liquid or solid‐phase extractions and the limits of quantification range from 50 to 0.005 ng/ml. The most important details of each analytical method are reported in Table 1.

**TABLE 1:** Summary of the analytical methods for grapiprant quantification reported in the literature

Reference	Clean‐up	Matrix	LOQ	Analytical method	Validated following FDA/EMA guideline
Rausch‐Derra and Rhodes (2016)	NA	Feline serum	NA	NA	NA
De Vito et al. (2017)	Liquid‐liquid extraction	Rabbit plasma	10 ng/ml	HPLC‐FL	Yes
Nagahisa and Okumura (2017)	Liquid‐liquid extraction	Canine plasma	NA	LC/MS/MS	NA
Heit et al. (2018)	NA	NA	NA	LC/MS/MS	NA
Knych et al. (2018)	Plasma deproteinization	Horse serum	0.005 ng/ml	LC‐MS/MS	Yes
	Solid‐phase extraction	Horse urine	0.1 ng/ml		
Cox et al. (2020)	Liquid‐liquid extraction	Horse plasma	50 ng/ml	HPLC‐FL	Yes
		Horse urine	50 ng/ml		
Łebkowska‐Wieruszewska, Barsotti, et al. (2017)	Liquid‐liquid extraction	Canine plasma	10 ng/ml	HPLC‐FL	Yes
Rausch‐Derra, Rhodes, et al. (2016)	NA	Canine serum	NA	LC/MS/MS	Yes
Łebkowska‐Wieruszewska, De Vito, et al. (2017)	Liquid‐liquid extraction	Feline plasma	1 ng/ml	HPLC‐FL	Yes
Baralla et al. (2018)	Liquid‐liquid extraction	Rabbit plasma	5 ng/ml	LC‐MS/MS	Yes
De Vito et al. (2016)	Liquid‐liquid extraction	Canine plasma	10 ng/ml	HPLC‐FL	Yes

Abbreviation: NA, not available.

## 4 PHARMACOKINETICS

The pharmacokinetics of grapirant have mainly been described in the canine species although a few pioneer studies have been published in cats, horses and rabbits (Table 2). Other unpublished studies (at the time of submission of this review) are ongoing in small ruminants and birds.

**TABLE 2:** Summary of the pharmacokinetic and safety studies published in the literature

Reference	*n*	Species	Drug formulation	Feed status	Route of administration	Dosage schedule	Dose	Safety data
Rausch‐Derra and Rhodes (2016)	24	Cats	Gelatine capsule	Fasted	PO	Daily for 28 days	3, 9, or 15 mg/kg	No adverse effects were detected at doses ≤15 mg/kg
De Vito et al. (2017)	12	New Zealand White rabbits	Solution in ethanol	Fasted	IV	Single dose	2 mg/kg	NA
Nagahisa and Okumura (2017)	12	Beagle dogs	Suspension in methylcellulose	Fasted	PO	Single dose	1, 3 and 10 mg/kg	No visible side effects
			Solution in sulfobutylether‐β‐cyclodextrin		IV		1 mg/kg	
Heit et al. (2018)	8	Mdr1‐deficient collies	Tablet	Fasted	PO	Daily for 28 days	~ 2 mg/kg	No visible side effects
Knych et al. (2018)	12	Thoroughbred horses	Tablet suspended in water	Fasted	PO	Single dose	2 mg/kg	No visible side effects
Cox et al. (2020)	6	Mares	Tablet suspended in water	Fasted	Via nasogastric tube	Single dose	2 mg/kg	No visible side effects
Łebkowska‐Wieruszewska, Barsotti, et al. (2017)	8	Labrador retriever dogs	Gelatine capsule	Fasted	PO	Single dose	2 mg/kg	No visible side effects
				Fed	PO		2 mg/kg	
			Ethanol solution		IV		0.5 mg/kg	
Rausch‐Derra, Rhodes, et al. (2016)	16	Beagle dogs	Tablet and suspension	Fasted	PO	Single dose	6 and 50 mg/kg	No severe side effects (mild gastrointestinal signs)
Łebkowska‐Wieruszewska, De Vito, et al. (2017)	6	European cats	Gelatine capsule	Fasted	PO	Single dose	2 mg/kg	No visible side effects
			Ethanol solution		IV		2 mg/kg	
Rausch‐Derra, Huebner, et al. (2016)	285	Beagle dogs with natural OA	Tablet	NA	PO	Daily for 28 days	0, 2 mg/kg	No visible side effects (drug‐related adverse events were mild and transient)
Rausch‐Derra et al. (2015)	36	Beagle dogs	Suspension in methylcellulose	Fed	PO	Daily for 9 months	0, 1, 6 and 50 mg/kg	Animals remain clinically normal

Abbreviation: NA, not assessed.

### 4.1 Dogs

A study in a tightly controlled research environment was conducted with Beagle dogs (Nagahisa & Okumura, 2017). Grapiprant was administered to animals in accordance with the exact body weight (methyl cellulose suspension of grapiprant at 1, 3 and 10 mg/kg PO and 1 mg/kg IV). The plasma concentration of grapiprant after 1, 3 and 10 mg/kg PO administration rapidly increased with *T*_max_ of 0.42–0.67 h. The plasma levels of grapiprant increased in a dose‐dependent manner but not in linear fashion (AUC_0–24_ were 1800, 7600 and 31,000 ng h/ml for 1, 3 and 10 mg/kg, respectively). This behaviour might be due to the small animal sample size of the study (3 animals for group). The serum protein binding of grapiprant was high (95%). The grapiprant *t*_1/2_ *_λz_* range was relatively broad 3.7–6.1 h. Surprisingly, the absolute bioavailability values were 62%, 90% and 110% at the doses of 1, 3 and 10 mg/kg, respectively. The apparent increase in F% with increasing doses was speculated to be due to saturation of efflux pumps and/or enzymatic metabolism.

The bioavailability of grapiprant after oral administration was estimated in fasted and fed Labrador retriever dogs (Łebkowska‐Wieruszewska, Barsotti, et al., 2017). The IV dose at 0.5 mg/kg, freshly dissolved in ethanol at 50 mg/ml, was administered to two animals. The plasma concentrations vs. time curves of the two dogs were almost identical. The oral dose was 2 mg/kg. This dose was prepared by micronizing the pure grapiprant powder manually in a mortar and putting the required dose into empty gelatine capsules. Animals (*n* = 6) received PO grapiprant after both fasting for 12 h overnight and in fed conditions. In this latter status, animals were fed one hour prior to drug administration. After oral administration of grapiprant to fed dogs, there was a delay in absorption, but this varied between dogs. The fed condition resulted in a reduced *C*_max_ (614 vs. 1598 ng/ml) and delayed *T*_max_ (3 vs. 1 h) compared to fasting dogs. This result differs from that presented by the pharmaceutical company (Anonymous, 2016b). The *t*_1/2_ *_λz_* was similar to that observed in the oral fasted and IV groups. The estimated absolute bioavailability in the fed group was 66.19% while it was almost twice that in fasting animals. Despite this large difference, the decrease in concentrations of grapiprant in plasma triggered by the fed condition mostly affected *C*_max_ but had negligible effects on the time when concentrations were greater than the theoretical canine minimal effective concentration (MEC) (Nagahisa & Okumura, 2017).

Sixteen Beagle dogs were randomized to receive single doses of either 6 or 50 mg/kg grapiprant as both suspension and tablet formulations within a cross‐over design with a 15‐day washout in order to evaluate its pharmacokinetics (Rausch‐Derra et al., 2016). Grapiprant rapidly achieved elevated concentrations in the sera, with mean *T*_max_ of <2 h regardless of formulation. Half‐lives ranged between approximately 3 and 11 h. As expected, the higher dose resulted in substantially higher *C*_max_ and AUC_last_ values compared to the lower dose. However, this increase did not correlate with the dose in a linear fashion as it had in the study of Nagahisa and Okumura (2017). A likely explanation for this discrepancy is the non‐linearity of clearance and volume of distribution with dose, as speculated by Łebkowska‐Wieruszewska, Barsotti, et al. (2017).

A short note reported the pharmacokinetics of grapiprant in Mdr1‐deficient collies (Heit et al., 2018). Eight adult collie dogs homozygous for the Mdr1 mutation were treated with approximately, but no less than, 2 mg/kg grapiprant orally once daily for 28 days after an overnight fast. Grapiprant was readily absorbed with a *C*_max_ of approximately 5000 ng/ml occurring at a median *T*_max_ of 1 h. Although no difference was seen in *t*_1/2_ *_λz_* (mean 4.6 h), the *C*_max_ and AUC (16,000 ng ×h/ml) were approximately fourfold and sixfold greater, respectively, than those reported in normal Beagles. It was concluded that Mdr1‐deficient dogs are expected to have higher AUC and *C*_max_ values compared to normal dogs treated with the same dose.

The main pharmacokinetic parameters of grapiprant reported in the above‐mentioned studies are summarized in Table 3.

**TABLE 3:** Main pharmacokinetic parameters of grapiprant found in the literature in different veterinary species

	*C*_max_ or *C*_0_	*T*_max_	*t*_1/2kel_	Cl	*V*_ss_	*F*
	ng/ml	h	h	ml/h/kg	ml/kg	%
Rausch‐Derra and Rhodes (2016)	D1, 1151	D1, 1.42	D1, 8.60	NA	NA	NA
	D2, 2730	D2, 1.83	D2, 8.45			
	D3, 2510*	D3, 1.17*	D3, 5.05*			
De Vito et al. (2017)	7086.9	NA	2.18	739.48	1258.26	NA
Nagahisa and Okumura (2017)	D1, 760	D1, 0.4	4.2	348	NA	D1, 62
	D2, 3600	D2, 0.58				D2, 90
	D3, 14000	D3, 0.67				D3, 110
Heit et al. (2018)	5000	1	4.6	NA	NA	NA
Knych et al. (2018)	31.1	1.5	5.86	NA	NA	NA
Cox et al. (2020)	106	0.5	NA	NA	NA	NA
Łebkowska‐Wieruszewska, Barsotti, et al. (2017)	Fasted, 1598	Fasted, 1.0	5.68	460	2480	Fasted, 111.9
	Fed, 614	Fed, 3.0				Fed, 66.19
Rausch‐Derra, Rhodes, et al. (2016)	D1, 3520–9210 (T)	D1, 1.0–2.0 (T)	D1, 1.95–5.42 (T)	NA	NA	NA
	2610–6150 (S)	1.0 (S)	1.64–9.04 (S)			
	D3, 94,800–114,000 (T)	D3, 1.0–2.0 (T)	D3, 2.23–7.76 (T)			
	46,000–91,900 (S)	1.0 (S)	4.03–5.19 (S)			
Łebkowska‐Wieruszewska, De Vito, et al. (2017)	625	1.33	5.48	173.2	918.5	39.62

Abbreviations: *C*_max_/*C*_0_, peak plasma concentration; *T*_max_, time of peak concentration; *t*_1/2kel_, terminal half‐life; Cl, plasma clearance; *V*_ss_, volume of distribution at the steady state; *F*, oral bioavailability. NA, not assessed. *, average between sexes. T, tablet; S, suspension. D1, lower dosage; D2, intermediate dosage; D3, higher dosage (for the dose details please refer to Table 2).

The variability in plasma concentrations and in some PK parameters found in the above‐mentioned studies might be due to the small animal sample size used. A remarkable result is that oral absorption of grapiprant is decreased by feeding (Łebkowska‐Wieruszewska, Barsotti, et al., 2017), this finding is not in line with the report issued by the pharmaceutical company (Anonymous, 2016a, 2016b). However, the instruction sheet of the drug (Anonymous, 2016b) does not recommend administration in the fed state. Indeed, it has been reported that the time range in which grapiprant plasma concentration is above the minimal effective concentration is similar in fasted and fed dogs (Łebkowska‐Wieruszewska, Barsotti, et al., 2017) without potential clinical implications. The same can be said for the plasma differences reported in Mdr1‐deficient vs. normal dogs. Although the differences in AUC values are significant, no adverse effect has been shown in Mdr1 dogs with 6 times higher grapiprant plasma concentrations (Heit et al., 2018).

### 4.2 Cats

The pharmacokinetics of grapiprant after 2 mg/kg administration via PO and IV (ethanol solution) routes in cats were assessed (Łebkowska‐Wieruszewska, De Vito, et al., 2017). Six healthy adult cats were used according to a 2 × 2, randomized cross‐over study design. Wide variability in plasma concentration of grapiprant was found after oral administration. Generally, oral absorption was rapid (mean *T*_max_ 1.33 h, range 1–2 h) reaching *C*_max_ in the range 490–750 ng/ml (median 625 ng/ml). The median *t*_1/2_ *_λz_* value (4.40 h) was similar to that found after IV administration. The PO bioavailability was 39.6%. The extraction ratio (Toutain & Bousquet‐Mélou, 2004) values in cats were in the range 1.5%–3.7%. These values are considered low and suggest that the contribution of the liver and kidney in the clearance of grapiprant might be limited.

The safety and toxicokinetic profiles associated with daily oral administration of grapiprant were evaluated (Rausch‐Derra & Rhodes, 2016). Cats (both gender) were randomly assigned (3 × group) to receive a placebo capsule or grapiprant at 3, 9 or 15 mg/kg, administered PO once daily for 28 days. Very broad variation was found in the pharmacokinetic parameters after the last grapiprant dose. *C*_max_ ranged from 683 to 4950 ng/ml with *T*_max_ within 1–4 h. Half‐lives ranged from 2 to 14 h. The comparison between the AUC after the first dose and that at the 27th indicated that no drug accumulation was likely. No apparent differences were reported in the pharmacokinetic parameters between sexes.

### 4.3 Horses

The drug concentrations and the pharmacokinetics of grapiprant in exercised Thoroughbred horses administered with a 2 mg/kg oral dose of grapiprant were described (Knych et al., 2018).

The *C*_max_, *T*_max_ and *t*_1/2_ *_λz_* were 31.9 ± 13.9 ng/ml, 1.5 ± 0.5 h and 5.86 ± 2.46 h, respectively. An oral dose of 2 mg/kg appeared not to achieve the MEC reported for dogs (Nagahisa & Okumura, 2017).

A recent study determined if the approved dose of grapiprant in dogs would produce measurable concentrations in urine and plasma of horses (Cox et al., 2020). Six mares were administered one dose of 2 mg/kg grapiprant via nasogastric tube. Grapiprant plasma concentrations ranged from 71 to 149 ng/ml with the mean peak concentration (106 ng/ml) occurring at 30 minutes. This study also reported that oral administration of grapiprant (2 mg/kg) in horses did not achieve the MEC reported for dogs (Nagahisa & Okumura, 2017).

### 4.4 Rabbits

The pharmacokinetics of grapiprant after 2 mg/kg IV administration were described (De Vito et al., 2017). Grapiprant plasma concentrations were detectable up to the 10‐h time point (concentration range 17–7495 ng/ml). Grapiprant was eliminated quite rapidly with a *t*_1/2_ *_λz_* value of 2.18 h. Clearance was 739.48 ml/h/kg with a low extraction ratio (range 7.7%–8.9%), and volume of distribution was large (2434.4 ml/kg).

## 5 PHARMACODYNAMICS

**FIGURE 2:**
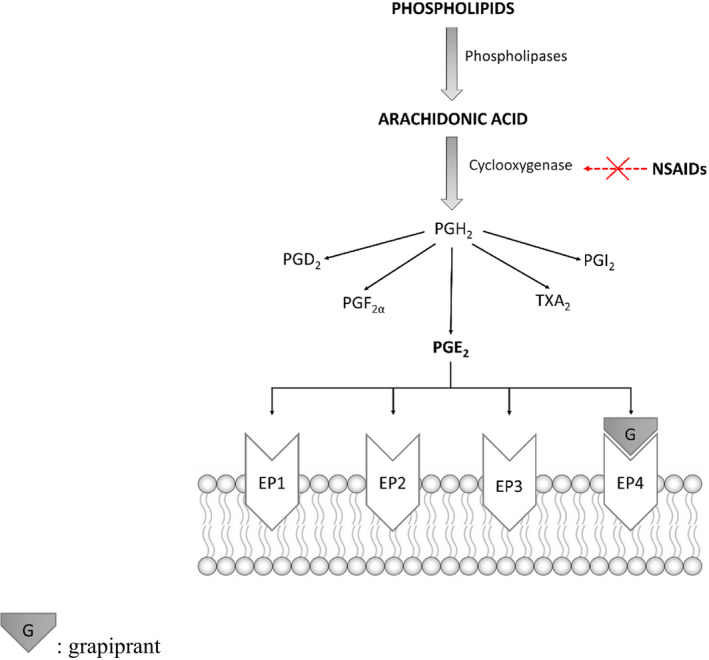
Prostaglandins biosynthesis and related receptors. Grapiprant target is highlighted

Grapiprant's mechanism of action targets the EP4 receptor (Figure 2). It does not inhibit the production of other prostanoids and does not interfere with the maintenance of numerous normal homeostatic functions, preventing the classic adverse effects associated with NSAIDs (Giorgi, 2015; Kirkby Shaw et al., 2016).

**FIGURE 3:**
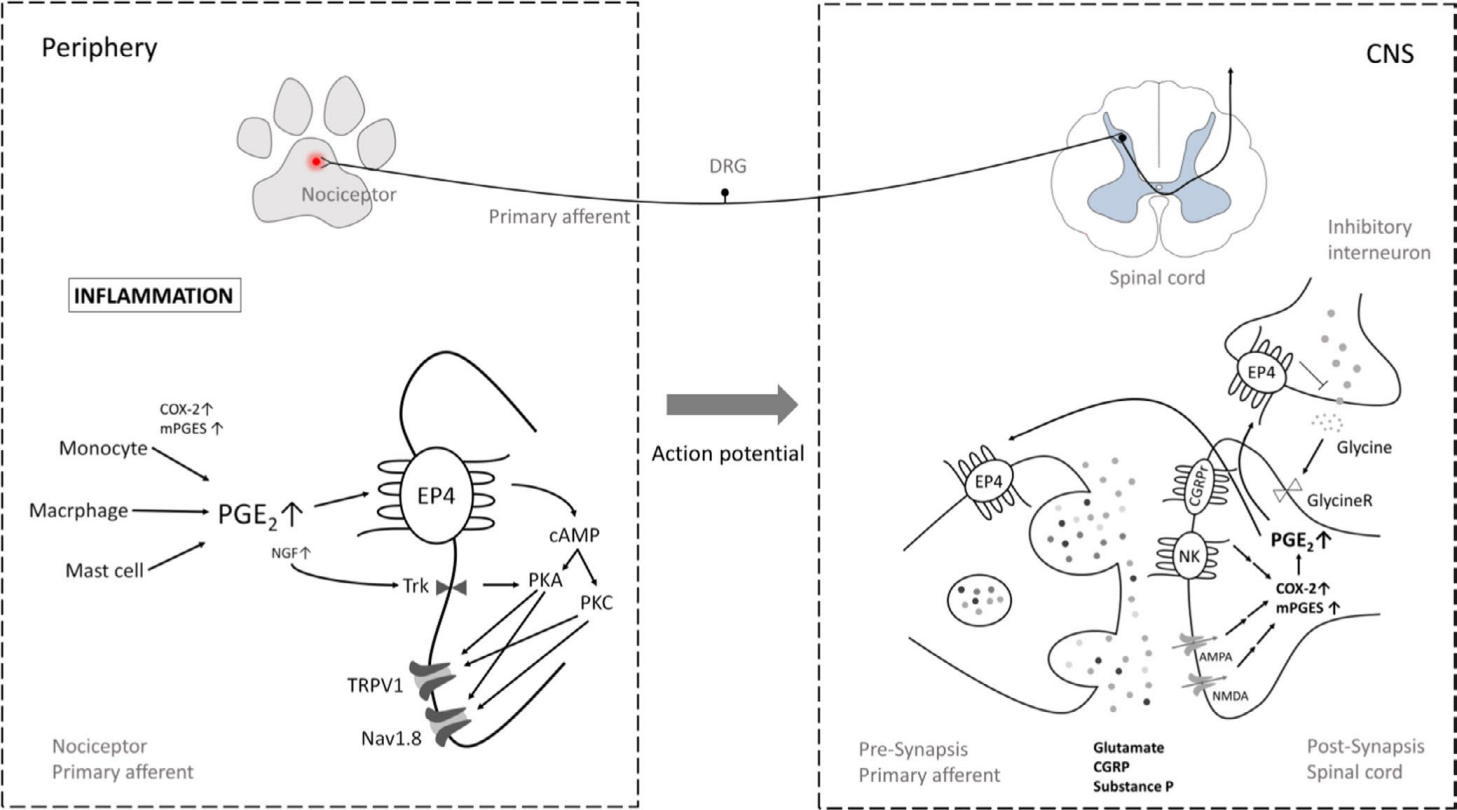
Brief overview of the nociceptive system (top panel) and the role of PGE2 and the EP4 receptor in inflammatory nociception on the molecular level (bottom panel). AMPA, α‐amino‐3‐hydroxy‐5‐methyl‐4‐isoxazolepropionic acid receptor; CGRP, calcitonin gene‐related protein; CGRPr, calcitonin gene‐related protein receptor; CNS, central nervous system; DRG, dorsal root ganglia; Nav1.8, voltage‐gated sodium channel; NK, neurokinin receptor; NMDA, N‐methyl‐D‐aspartate receptor; PKA, protein kinase A; PKC, protein kinase C; Trk, Tropomyosin receptor kinase A (NGF receptor)

EP4, along with EP1, EP2, EP3, is one of the target receptors for PGE2 (Woodward et al., 2011), is the most abundant PG in synovia and is a key mediator of inflammation and pain (Yokoyama et al., 2013). The EP4 receptor has been shown to regulate inflammatory nociception at the peripheral and CNS level. Particularly in inflammatory pain, a number of inflammatory mediators in the periphery such as PGs, nerve growth factor and bradykinin activate and/or sensitize neurons (Basbaum et al., 2009; Mizumura et al., 2009). PGE2 is a typical inflammatory mediator released after induction of COX‐2 in injured tissue and cells, mostly immune cells such as mast cells, basophils or macrophages, which accumulate in the area of injury. PGE2 binds to EP receptors with subsequent activation of intracellular signalling pathways resulting in an increase in intracellular cAMP and thus activation of protein kinases. The latter leads to phosphorylation of a number of target proteins including TRPV1 channels, voltage‐gated Na+ or T‐type calcium channels which contribute to sensitization mechanisms in the peripheral nervous system (Kawabata, 2011). This sensitization also occurs in the CNS by gene regulation in the pre‐ and post‐synaptic neurons (Kuner, 2010). COX‐2 is constitutively expressed in neuronal and glial cells of the CNS and upregulated after ongoing nociceptive stimulation associated with augmented PG synthesis which leads to increased neurotransmitter release (Ferreira & Lorenzetti, 1996; Nishihara et al., 1995), enhanced calcium concentrations and activation of a number of pain‐related genes (Figure 3). The consequence of peripheral and central sensitization is an increased sensitivity towards nociceptive stimuli (hyperalgesia) or a nociceptive response to non‐noxious stimulation (allodynia).

EP4 plays a crucial role in the development of acute and chronic pain and in inflammation and is therefore one of the most important executors of PGE2 effects in these processes, indicating that this receptor might also be a good target for the development of new anti‐inflammatory and analgesic drugs. Peripheral PGE2 prolongs the sensitization of nociceptive dorsal root ganglion neurons possibly by facilitating the synthesis and anterograde axonal trafficking of EP4 receptors (Kawahara et al., 2015; Lin et al., 2006; Schiffmann et al., 2014; St‐Jacques & Ma, 2014).

The canine EP4 receptor has approximately 90% homology to the human receptor (Castleberry et al., 2001), and it is a Gsα‐ (that stimulates the cAMP‐dependent pathway by activating adenylyl cyclase) and Giα‐ (inhibits the cAMP‐dependent pathway by inhibiting adenylyl cyclase) coupled protein (Yokoyama et al., 2013). EP4 signalling plays a variety of roles through stimulating adenylate cyclase leading to elevated levels of intracellular cAMP (Yokoyama et al., 2013). The EP4 receptor has been showed to be specifically involved in the pain and inflammation associated with experimentally induced arthritis in rodents (Boyd et al., 2011; Chen et al., 2010; Clark et al., 2008; Kabashima et al., 2007; Lin et al., 2006; McCoy et al., 2002; Nakao et al., 2007; Southall & Vasko, 2001; St‐Jacques & Ma, 2013).

Grapiprant binds to human and rat EP4 receptors with high affinity (Nakao et al., 2007) and it has been found to have a similar potency in humans (IC_50_ 14 ± 3.9 nM; Ki 13 ± 4 nM), rats (IC_50_ 27 ± 1.3 nM; Ki 20 ± 1 nM) (Nakao et al., 2007) and in dogs (Nagahisa & Okumura, 2017). Grapiprant was found to displace [3H]‐PGE2 (1 nM) binding to dog recombinant EP4 receptor in a concentration‐dependent manner with an IC_50_ value of 35 ± 3.9 nM and with a Ki value of 24 ± 2.7 nM (Nagahisa & Okumura, 2017).

### 5.1 Dogs

The oral dose predicted to be efficacious in dogs was computed based on the comparison between the affinity of grapiprant to dog, rat and human EP4 receptors. It resulted in a range of 1.3 and 1.7 mg/kg once a day (Nagahisa & Okumura, 2017) while the approved clinical dose was set at 2 mg/kg once a day (Rausch‐Derra, Huebner, et al., 2016). The calculated MEC range and efficacious AUC_0‐24_ were 114–164 ng/ml and 3936 ng h/ml, respectively (Nagahisa & Okumura, 2017).

The efficacy and safety of grapiprant in dogs with natural OA were assessed (Rausch‐Derra, Huebner, et al., 2016). Two hundred and eighty‐five client‐owned dogs with OA were enrolled and treated with grapiprant or placebo with 262 cases (*N* = 131 in each group) available for the effectiveness analysis. In this prospective, randomized, masked, placebo‐controlled study, dogs were treated daily with grapiprant (2 mg/kg) PO or placebo. Owners completed an evaluation using the Canine Brief Pain Inventory (CBPI) on days 0, 7, 14, 21 and 28. Success was defined as improvement in the CBPI. Veterinary assessments were made on screening and days 14 and 28. Safety was evaluated by physical examination, evaluation of clinical pathology results and owner observations. Grapiprant treatment significantly improved pain compared to placebo on day 28 (48.1 and 31.3% treatment successes, respectively; *p* = 0.0315). The pain interference score and pain severity score improved in the grapiprant group compared to placebo (*p* = 0.0029 and 0.0022, respectively). Veterinary assessments were significantly better in the grapiprant‐treated dogs (*p* = 0.0086).

A randomized, two‐sequence, assessor‐blinded study involving two separate experiments measured the potency and persistence of acute pain control over 24 h resulting from a single oral dose of either firocoxib (5.7–8.5 mg/kg) or grapiprant (1.5–2.9 mg/kg) in an acute canine arthritis urate crystal model in 18 Beagle dogs (de Salazar Alcalá et al., 2019). The analgesic efficacy of each treatment was assessed on the basis of the vertical force (VF) applied by each dog's hind limb, measured with a force plate and on visual lameness (VL) scores (from 0 to 5) calculated for each dog, on each experiment and at each time point. In both the experiments, firocoxib was superior to grapiprant. Grapiprant was shown to produce an effect not statistically different from the control.

The ability of a proprietary antagonist of the EP 4 receptor (4 mg/kg), grapiprant (2 mg/kg) and carprofen (4.4 mg/kg) to attenuate lameness attributable to urate‐induced synovitis was investigated in five dogs (Budsberg et al., 2019). Measurements of vertical ground reaction forces and clinical lameness scores were used to assess the analgesic activities of the different compounds. Carprofen was the most effective treatment for attenuating lameness induced by injection of sodium urate, and grapiprant was the least effective treatment.

Grapiprant is a newly introduced drug, and so reports of testing under laboratory and field conditions have been limited to studies that are required for regulatory approvals (Rausch‐Derra, Huebner, et al., 2016). Surprisingly two experimental (Budsberg et al., 2019; de Salazar Alcalá et al., 2019) studies show that grapiprant, a drug with presumptive advantages, has less efficacy than established treatments. It should be noted that the experimental animal model used (intra‐articular injection of sodium urate in the stifle joints of dogs) produces acute pain while grapiprant is approved for chronic pain. It is noteworthy that the same experimental model (adjuvant‐induced arthritis) was used in rats to demonstrate the efficacy of grapiprant (Anonymous, 2016a). Several studies have correlated the pain relief produced with this model with those reported in clinical trials attributable to OA in dogs (Borer et al., 2017; Cross et al., 1997; Doig et al., 2000). Unfortunately, only a single clinical trial has been carried out to evaluate the real clinical efficacy of this active ingredient (Rausch‐Derra, Huebner, et al., 2016). Although results in general are encouraging, the efficacy compared to the placebo had a *p* value of 0.044, which is barely significant, and the efficacy response was only 41%–46%. Additional trials are warranted to confirm its efficacy.

### 5.2 Rabbits

The thermal antinociceptive effect of grapiprant in the induced inflammatory pain model in the rabbit after a single IV injection of 2 mg/kg was evaluated and compared with that generated by 0.5 mg/kg meloxicam injected subcutaneously (De Vito et al., 2017). An infrared thermal stimuli was applied to the plantar surface of the rabbits' hindlimbs to evaluate the thermal withdrawal latency (TWL). The thermal antinociceptive effect was expressed as maximum possible response. The grapiprant‐treated animals showed a significant increase in TWL from 1 h and up to 10 h after drug administration compared to the control and TWL was higher than meloxicam in the range 1–2 h and at 10 h. The difference in efficacy in the initial hours was supposed to be due to the different routes of administration of the two drugs. The increased efficacy shown at 10 h was speculated to be due to the different doses of drugs administered, to a wider counter‐clockwise hysteresis loop or to a potential active metabolite (never detected) of grapiprant that might have prolonged the treatment effect. Also the different mechanisms of action of the two drugs might have played a role in this effect. The antinociceptive effect of grapiprant and meloxicam was not found to be statistically different in the time range 4–8 h.

## 6 SAFETY PROFILE

### 6.1 Dogs

None of the studies administering a single oral dose in dogs (even larger that the clinical one) reported any visible side effect (Łebkowska‐Wieruszewska, Barsotti, et al., 2017; Nagahisa & Okumura, 2017).

The safety of long‐term, daily oral administration of grapiprant was tested (Rausch‐Derra et al., 2015). Thirty‐six dogs were randomly assigned to four groups (8 dogs/group) that received grapiprant via oral gavage at 0, 1, 6 or 50 mg/kg, once a day for 9 months. All dogs received daily ophthalmologic, ECG, and laboratory evaluations before treatment began (baseline) and periodically afterwards. Dogs were euthanized at the end of the study for necropsy and histologic evaluation. All dogs remained clinically normal during treatment, with no apparent changes in appetite or demeanour. Emesis and soft or mucoid faeces that occasionally contained blood were observed in all groups, although these findings were more common in dogs that received grapiprant. In general, clinicopathologic findings remained within baseline ranges. Drug‐related changes in serum total protein and albumin concentrations were detected, but differences were small and resolved during recovery. No drug‐related gross or microscopic pathological changes were detected in tissue samples except mild mucosal regeneration in the ileum of one dog in the 50 mg/kg group. These findings suggested that long‐term administration of grapiprant is safe in dogs. It might be suggested that if any other NSAID was administered once daily for 9 months at 25 times its clinical dose, the consequences would have been more serious than those reported for grapiprant.

To the best of Authors' knowledge, no study comparing the adverse effects of grapiprant and any other NSAID is available. However, the pharmaceutical company reports that a dog group (*n* = 94) treated with 2 mg/kg once daily dose had similar incidence rates for adverse events (diarrhoea, vomiting and inappetence) to the placebo group (*n* = 88) (Anonymous, 2016a).

When an oral dose of 2 mg/kg was administered daily for 28 days to normal Beagles and Mdr1‐deficient dogs, despite grapiprant exposure being greater in these latter animals, no differences in general health, clinical sign or body weight were found (Heit et al., 2018).

### 6.2 Cats

A single oral dose of grapirant at 2 mg/kg (IV and PO) did not produce visible side effects in cats (Łebkowska‐Wieruszewska, De Vito, et al., 2017).

The safety profile was evaluated after daily PO administration of 3, 9 or 15 mg/kg over a period of 4 weeks (Rausch‐Derra & Rhodes, 2016). Grapiprant was well tolerated, and no adverse effects were detected at doses ≤15 mg/kg. No significant effects on body weight, food consumption, clinicopathologic variables, or gross or histologic necropsy findings were noted.

### 6.3 Other animal species

Rabbits after single IV (0.5 mg/kg) and PO (2 mg/kg) dose and horses after single PO dose (2 mg/kg) did not report any significant adverse effects (Cox et al., 2020; De Vito et al., 2017; Knych et al., 2018).

## 7 CONCLUSION

A number of studies have been carried out to assess diverse analytical methods for grapiprant quantification, its pharmacokinetics, pharmacodynamics and safety especially in the canine species. The pharmacokinetic profile seems well characterised in dogs. Although some data are conflicting (oral bioavailability fed vs. fasted), the differences in plasma concentrations might not generate appreciable variation in clinical efficacy. In terms of efficacy, grapiprant is barely better than placebo and inferior to other traditional NSAIDs. It may be an alternative to traditional NSAIDs because of its alternative mode of action, however, further clinical studies are warranted to clarify this point.

## CONFLICT OF INTEREST

Authors declare no conflict of interest.

## AUTHOR CONTRIBUTIONS

MG and IS conceived of the presented idea. Both authors read and discussed the literarure and contributed to the final manuscript.

## Data Availability

Please contact the author for any requests for access to the data.

## References

[jvp12983-bib-0001] Anonymous (2016a). NADA 141‐455 GALLIPRANT. https://animaldrugsatfda.fda.gov/adafda/app/search/public/document/downloadFoi/941

[jvp12983-bib-0002] Anonymous (2016b). Galliprant prescribing information. Aratana Therapeutics. https://files.brief.vet/2018‐07/Galliprant%20Prescribing%20Information_Elanco.pdf

[jvp12983-bib-0003] Baralla, E. , Vito, V. D. , Varoni, M. V. , Giorgi, M. , & Demontis, M. (2018). Novel LC‐MS/MS method for CJ‐023423 (grapiprant) determination in rabbit plasma. American Journal of Animal and Veterinary Sciences, 13, 45–50. 10.3844/ajavsp.2018.45.50

[jvp12983-bib-0004] Basbaum, A. I. , Bautista, D. M. , Scherrer, G. , & Julius, D. (2009). Cellular and molecular mechanisms of pain. Cell, 139, 267–284. 10.1016/j.cell.2009.09.028 19837031 PMC2852643

[jvp12983-bib-0005] Borer, L. R. , Seewald, W. , Peel, J. E. , & King, J. N. (2017). Evaluation of the dose‐response relationship of oral robenacoxib in urate crystal‐induced acute stifle synovitis in dogs. Journal of Veterinary Pharmacology and Therapeutics, 40, 148–157. 10.1111/jvp.12348 27493016

[jvp12983-bib-0006] Boyd, M. J. , Berthelette, C. , Chiasson, J. F. , Clark, P. , Colucci, J. , Denis, D. , Han, Y. , Lévesque, J. F. , Mathieu, M. C. , Stocco, R. , Therien, A. , Rowland, S. , Wrona, M. , & Xu, D. (2011). A novel series of potent and selective EP4 receptor ligands: facile modulation of agonism and antagonism. Bioorganic & Medicinal Chemistry Letters, 21, 484–487. 10.1016/j.bmcl.2010.10.106 21126875

[jvp12983-bib-0007] Budsberg, S. C. , Kleine, S. A. , Norton, M. M. , & Sandberg, G. S. (2019). Comparison of two inhibitors of E‐type prostanoid receptor four and carprofen in dogs with experimentally induced acute synovitis. American Journal of Veterinary Research, 80, 1001–1006. 10.2460/ajvr.80.11.1001 31644340

[jvp12983-bib-0008] Castleberry, T. A. , Lu, B. , Smock, S. L. , & Owen, T. A. (2001). Molecular cloning and functional characterization of the canine prostaglandin E2 receptor EP4 subtype. Prostaglandins & Other Lipid Mediators, 65, 167–187. 10.1016/S0090-6980(01)00129-0 11444589

[jvp12983-bib-0009] Chen, Q. , Muramoto, K. , Masaaki, N. , Ding, Y. , Yang, H. , Mackey, M. , Li, W. , Inoue, Y. , Ackermann, K. , Shirota, H. , Matsumoto, I. , Spyvee, M. , Schiller, S. , Sumida, T. , Gusovsky, F. , & Lamphier, M. (2010). A novel antagonist of the prostaglandin E(2) EP(4) receptor inhibits Th1 differentiation and Th17 expansion and is orally active in arthritis models. British Journal of Pharmacology, 160, 292–310. 10.1111/j.1476-5381.2010.00647.x 20423341 PMC2874852

[jvp12983-bib-0010] Clark, P. , Rowland, S. E. , Denis, D. , Mathieu, M. C. , Stocco, R. , Poirier, H. , Burch, J. , Han, Y. , Audoly, L. , Therien, A. G. , & Xu, D. (2008). MF498 [N‐{[4‐(5,9‐Diethoxy6‐oxo‐6,8‐dihydro‐7H‐pyrrolo[3,4‐g]quinolin‐7‐yl)‐3‐methylbenzyl]sulfonyl}‐2‐(2‐methoxyphenyl)acetamide], a selective E prostanoid receptor 4 antagonist, relieves joint inflammation and pain in rodent models of rheumatoid and osteoarthritis. The Journal of Pharmacology and Experimental Therapeutics, 325, 425–434. 18287210 10.1124/jpet.107.134510

[jvp12983-bib-0011] Cox, S. , Sommardahl, C. , Fortner, C. , Davis, R. , Bergman, J. , & Doherty, T. (2020). Determination of grapiprant plasma and urine concentrations in horses. Veterinary Anaesthesia and Analgesia, 47, 705–709. 10.1016/j.vaa.2020.04.006 32439238

[jvp12983-bib-0012] Cross, A. R. , Budsberg, S. C. , & Keefe, T. J. (1997). Kinetic gait analysis assessment of meloxicam efficacy in a sodium urate‐induced synovitis model in dogs. American Journal of Veterinary Research, 58, 626–631. 9185970

[jvp12983-bib-0013] de Salazar Alcalá, A. G. , Gioda, L. , Dehman, A. , & Beugnet, F. (2019). Correction to: Assessment of the efficacy of firocoxib (Previcox^®^) and grapiprant (Galliprant^®^) in an induced model of acute arthritis in dogs. BMC Veterinary Research, 15(1), 347. 10.1186/s12917-019-2116-1 31619253 PMC6796375

[jvp12983-bib-0014] De Vito, V. , Saba, A. , Lee, H. K. , Owen, H. , Poapolathep, A. , & Giorgi, M. (2016). Detection and quantification of the selective EP4 receptor antagonist CJ‐023423 (grapiprant) in canine plasma by HPLC with spectrofluorimetric detection. Journal of Pharmaceutical and Biomedical Analysis, 118, 251–258. 10.1016/j.jpba.2015.11.004 26580822

[jvp12983-bib-0015] De Vito, V. , Salvadori, M. , Poapolathep, A. , Owen, H. , Rychshanova, R. , & Giorgi, M. (2017). Pharmacokinetic/pharmacodynamic evaluation of grapiprant in a carrageenan‐induced inflammatory pain model in the rabbit. Journal of Veterinary Pharmacology and Therapeutics, 40, 468–475. 10.1111/jvp.12380 27925221

[jvp12983-bib-0016] Doig, P. A. , Purbrick, K. A. , Hare, J. E. , & McKeown, D. B. (2000). Clinical efficacy and tolerance of meloxicam in dogs with chronic osteoarthritis. The Canadian Veterinary Journal, 41, 296–300. 10769766 PMC1476158

[jvp12983-bib-0017] Ferreira, S. H. , & Lorenzetti, B. B. (1996). Intrathecal administration of prostaglandin E2 causes sensitization of the primary afferent neuron via the spinal release of glutamate. Inflammation Research, 45, 499–502. 10.1007/BF02311085 8912014

[jvp12983-bib-0018] Giorgi, M. (2012). Veterinary pharmacology: Is it still pharmacology Cinderella? Clinical and Experimental Pharmacology, 2, 1003–1004.

[jvp12983-bib-0019] Giorgi, M. (2015). CJ‐023,423 (Grapiprant) a potential novel active compound with antihyperalgetic properties for veterinary patients. American Journal of Animal and Veterinary Sciences, 10, 53–56. 10.3844/ajavsp.2015.53.56

[jvp12983-bib-0020] Giorgi, M. , Saccomanni, G. , Del Carlo, S. , Manera, C. , & Lavy, E. (2012). Pharmacokinetics of intravenous and intramuscular parecoxib in healthy beagles. Veterinary Journal, 193, 246–250. 10.1016/j.tvjl.2011.11.005 22130459

[jvp12983-bib-0021] Heit, M. C. , Stallons, L. J. , & Brossard, P. (2018). Tolerance and pharmacokinetics of Galliprant™ administered orally to Mdr1‐deficient collies. Session 18: Advances in companion animal medicine. Journal of Veterinary Pharmacology and Therapeutics, 41, 61–63.

[jvp12983-bib-0022] Johnston, S. A. , McLaughlin, R. M. , & Budsberg, S. C. (2008). Nonsurgical management of osteoarthritis in dogs. Veterinary Clinics of North America: Small Animal Practice, 38, 1449–1470. 10.1016/j.cvsm.2008.08.001 18954692

[jvp12983-bib-0023] Kabashima, K. , Nagamachi, M. , Honda, T. , Nishigori, C. , Miyachi, Y. , Tokura, Y. , & Narumiya, S. (2007). Prostaglandin E2 is required for ultraviolet B‐induced skin inflammation via EP2 and EP4 receptors. Laboratory Investigation, 87, 49–55. 10.1038/labinvest.3700491 17075575

[jvp12983-bib-0024] Kawabata, A. (2011). Prostaglandin E2 and pain–an update. Biological and Pharmaceutical Bulletin, 34, 1170–1173. 10.1248/bpb.34.1170 21804201

[jvp12983-bib-0025] Kawahara, K. , Hohjoh, H. , Inazumi, T. , Tsuchiya, S. , & Sugimoto, Y. (2015). Prostaglandin E2‐induced inflammation: relevance of prostaglandin E receptors. Biochimica Et Biophysica Acta, 1851, 414–421. 10.1016/j.bbalip.2014.07.008 25038274

[jvp12983-bib-0026] Kim, T. W. , & Giorgi, M. (2013). A brief overview of the coxib drugs in the veterinary field. American Journal of Animal and Veterinary Science, 8, 89–97. 10.3844/ajavsp.2013.89.97

[jvp12983-bib-0027] Kirkby Shaw, K. , Rausch‐Derra, L. C. , & Rhodes, L. (2016). Grapiprant: An EP4 prostaglandin receptor antagonist and novel therapy for pain and inflammation. Veterinary Medicine and Science, 2, 3–9. 29067176 10.1002/vms3.13 PMC5645826

[jvp12983-bib-0028] Knych, H. K. , Seminoff, K. , & McKemie, D. S. (2018). Detection of grapiprant following oral administration to exercised Thoroughbred horses. Drug Test Analysis, 10, 1237–1243. 10.1002/dta.2378 29575649

[jvp12983-bib-0029] Kuner, R. (2010). Central mechanisms of pathological pain. Nature Medicine, 16, 1258–1266. 10.1038/nm.2231 20948531

[jvp12983-bib-0030] Łebkowska‐Wieruszewska, B. , Barsotti, G. , Lisowski, A. , Gazzano, A. , Owen, H. , & Giorgi, M. (2017). Pharmacokinetics and estimated bioavailability of grapiprant, a novel selective prostaglandin E2 receptor antagonist, after oral administration in fasted and fed dogs. New Zealand Veterinary Journal, 65(1), 19–23. 27691904 10.1080/00480169.2016.1241727

[jvp12983-bib-0031] Łebkowska‐Wieruszewska, B. , De Vito, V. , Owen, H. , Poapholatep, A. , & Giorgi, M. (2017). Pharmacokinetics of grapiprant, a selective EP4 prostaglandin PGE2 receptor antagonist, after 2 mg/kg oral and i.v. administrations in cats. Journal of Veterinary Pharmacology and Therapeutics, 40(6), e11–e15. 28459136 10.1111/jvp.12414

[jvp12983-bib-0033] Lin, C. R. , Amaya, F. , Barrett, L. , Wang, H. , Takada, J. , Samad, T. A. , & Clifford, J. W. (2006). Prostaglandin E2 receptor EP4 contributes to inflammatory pain hypersensitivity. Journal of Pharmacology and Experimental Therapy, 319, 1096–1103. 10.1124/jpet.106.105569 16966471

[jvp12983-bib-0034] McCoy, J. M. , Wicks, J. R. , & Audoly, L. P. (2002). The role of prostaglandin E2 receptors in the pathogenesis of rheumatoid arthritis. The Journal of Clinical Investigation, 110, 651–658. 10.1172/JCI0215528 12208866 PMC151107

[jvp12983-bib-0035] McLaughlin, R. (2000). Management of chronic osteoarthritic pain. Veterinary Clinics of North America: Small Animal Practice, 30, 933–949. 10.1016/S0195-5616(08)70016-0 10932834

[jvp12983-bib-0036] Mizumura, K. , Sugiura, T. , Katanosaka, K. , Banik, R. K. , & Kozaki, Y. (2009). Excitation and sensitization of nociceptors by bradykinin: what do we know? Experimental Brain Research, 196, 53–65. 10.1007/s00221-009-1814-5 19396590

[jvp12983-bib-0037] Nagahisa, A. , & Okumura, T. (2017). Pharmacology of grapiprant, a novel EP4 antagonist: Receptor binding, efficacy in a rodent postoperative pain model, and a dose estimation for controlling pain in dogs. Journal of Veterinary Pharmacology and Therapeutics, 40, 285–292. 10.1111/jvp.12349 27597397

[jvp12983-bib-0038] Nakao, K. , Murase, A. , Ohshiro, H. , Okumura, T. , Taniguchi, K. , Murata, Y. , Masuda, M. , Kato, T. , Okumura, Y. , & Takada, J. (2007). CJ‐023,423, a novel, potent and selective prostaglandin EP4 receptor antagonist with antihyperalgesic properties. Journal of Pharmacology and Experimental Therapeutics, 322, 686–694. 10.1124/jpet.107.122010 17495127

[jvp12983-bib-0039] Nishihara, I. , Minami, T. , Watanabe, Y. , Ito, S. , & Hayaishi, O. (1995). Prostaglandin E2 stimulates glutamate release from synaptosomes of rat spinal cord. Neuroscience Letters, 196, 57–60. 10.1016/0304-3940(95)11839-O 7501257

[jvp12983-bib-0040] Rausch‐Derra, L. C. , Huebner, M. , & Rhodes, L. (2015). Evaluation of the safety of long‐term, daily oral administration of grapiprant, a novel drug for treatment of osteoarthritic pain and inflammation, in healthy dogs. American Journal of Veterinary Research, 76, 853–859. 10.2460/ajvr.76.10.853 26413822

[jvp12983-bib-0041] Rausch‐Derra, L. C. , Huebner, M. , Wofford, J. , & Rhodes, L. A. (2016). A prospective, randomized, masked, placebo‐controlled multisite clinical study of grapiprant, an EP4 prostaglandin receptor antagonist (PRA), in dogs with osteoarthritis. Journal of Veterinary Internal Medicine, 30, 756–763. 27075237 10.1111/jvim.13948 PMC4913586

[jvp12983-bib-0042] Rausch‐Derra, L. C. , & Rhodes, L. (2016). Safety and toxicokinetic profiles associated with daily oral administration of grapiprant, a selective antagonist of the prostaglandin E2 EP4 receptor, to cats. American Journal of Veterinary Research, 77, 688–692. 27347820 10.2460/ajvr.77.7.688

[jvp12983-bib-0043] Rausch‐Derra, L. C. , Rhodes, L. , Freshwater, L. , & Hawks, R. (2016). Pharmacokinetic comparison of oral tablet and suspension formulations of grapiprant, a novel therapeutic for the pain and inflammation of osteoarthritis in dogs. Journal of Veterinary Pharmacology and Therapeutics, 39, 566–571. 10.1111/jvp.12306 27027634

[jvp12983-bib-0044] Rychel, J. K. (2010). Diagnosis and treatment of osteoarthritis. Top Companion Animal Medicine, 25, 20–25. 10.1053/j.tcam.2009.10.005 20188335

[jvp12983-bib-0045] Schiffmann, S. , Weigert, A. , Mannich, J. , Eberle, M. , Birod, K. , Häussler, A. , Ferreiros, N. , Schreiber, Y. , Kunkel, H. , Grez, M. , Weichand, B. , Brüne, B. , Pfeilschifter, W. , Nüsing, R. , Niederberger, E. , Grösch, S. , Scholich, K. , & Geisslinger, G. (2014). PGE2/EP4 signaling in peripheral immune cells promotes development of experimental autoimmune encephalomyelitis. Biochemical Pharmacology, 87, 625–635. 10.1016/j.bcp.2013.12.006 24355567

[jvp12983-bib-0047] Southall, M. D. , & Vasko, M. R. (2001). Prostaglandin receptor subtypes, EP3C and EP4, mediate the prostaglandin E2‐induced cAMP production and sensitization of sensory neurons. The Journal of Biological Chemistry, 276, 16083–16091. 10.1074/jbc.M011408200 11278900

[jvp12983-bib-0048] St‐Jacques, B. , & Ma, W. (2013). Prostaglandin E2/EP4 signalling facilitates EP4 receptor externalization in primary sensory neurons in vitro and in vivo. Pain, 154, 313–323. 10.1016/j.pain.2012.11.005 23265688

[jvp12983-bib-0049] St‐Jacques, B. , & Ma, W. (2014). Peripheral prostaglandin E2 prolongs the sensitization of nociceptive dorsal root ganglion neurons possibly by facilitating the synthesis and anterograde axonal trafficking of EP4 receptors. Experimental Neurology, 261, 354–366. 10.1016/j.expneurol.2014.05.028 24910202

[jvp12983-bib-0050] Toutain, P. L. , & Bousquet‐Mélou, A. (2004). Plasma clearance. Journal of Veterinary Pharmacology and Therapeutics, 27, 415–425. 10.1111/j.1365-2885.2004.00605.x 15601437

[jvp12983-bib-0051] Woodward, D. F. , Jones, R. L. , & Narumiya, S. (2011). International Union of basic and clinical pharmacology. LXXXIII: classification of prostanoid receptors, updating 15 years of progress. Pharmacological Reviews, 63, 471–538. 10.1124/pr.110.003517 21752876

[jvp12983-bib-0052] Yokoyama, U. , Iwatsubo, K. , Umemura, M. , Fujita, T. , & Ishikawa, Y. (2013). The Prostanoid EP4 receptor and its signaling pathway. Pharmacological Reviews, 65, 1010–1052. 10.1124/pr.112.007195 23776144

